# Immunogenic Proteins of Group B Streptococcus—Potential Antigens in Immunodiagnostic Assay for GBS Detection

**DOI:** 10.3390/pathogens11010043

**Published:** 2021-12-31

**Authors:** Anna Dobrut, Monika Brzychczy-Włoch

**Affiliations:** Department of Molecular Medical Microbiology, Faculty of Medicine, Medical College, Jagiellonian University, 31-121 Krakow, Poland; anna.dobrut@uj.edu.pl

**Keywords:** *Streptococcus agalactiae*, Group B Streptococcus, immunogenic proteins, immunodiagnostics, proteomics, newborn infections, GBS carriage, biomarkers, innovative immunoassays

## Abstract

*Streptococcus agalactiae* (Group B Streptococcus, GBS) is an opportunistic pathogen, which asymptomatically colonizes the gastrointestinal and genitourinary tract of up to one third of healthy adults. Nevertheless, GBS carriage in pregnant women may lead to several health issues in newborns causing life threatening infection, such as sepsis, pneumonia or meningitis. Recommended GBS screening in pregnant women significantly reduced morbidity and mortality in infants. Nevertheless, intrapartum antibiotic prophylaxis, recommended following the detection of carriage or in case of lack of a carriage test result for pregnant women who demonstrate certain risk factors, led to the expansion of the adverse phenomenon of bacterial resistance to antibiotics. In our paper, we reviewed some immunogenic GBS proteins, i.e., Alp family proteins, β protein, Lmb, Sip, BibA, FsbA, ScpB, enolase, elongation factor Tu, IMPDH, and GroEL, which possess features characteristic of good candidates for immunodiagnostic assays for GBS carriage detection, such as immunoreactivity and specificity. We assume that they can be used as an alternative diagnostic method to the presently recommended bacteriological cultivation and MALDI.

## 1. Introduction

*Streptococcus agalactiae* (Group B Streptococcus, GBS) is a β-hemolytic, Gram-positive bacterium, which colonizes the gastrointestinal and genitourinary tract of up to 30% of healthy adults [1]. Each year, over 21 million pregnant women worldwide are colonized with GBS, which includes approximately 18% of pregnancies [2]. This opportunistic pathogen can cause a life-threatening infection in newborns, which most often takes the form of sepsis, pneumonia and meningitis, and the first two are more common for early onset GBS disease (EOD, EOGBSD). EOD appears in the first week of life, however, the vast majority of cases concerns the first 24 h and it is a consequence of an infection acquired during natural childbirth from a GBS-colonized mother [1]. In the early 1970s, mortality in this group was very high, and even reached 55% in newborns diagnosed with GBS infection [3].

In response to this threat, in the 1990s, the American College of Obstetricians and Gynecologists (ACOG) and Centers for Disease Control and Prevention (CDC) developed guidelines to minimize this dangerous phenomenon, which led to a reduction in morbidity in newborns of over 80%, and presently the incidence of the disease caused by GBS reaches 0.23/1000 live births [4,5,6]. It included screening recommendations for women between the 36th (36-0/7) and 37th (37-0/7) week of pregnancy, by taking swabs from the vaginal introitus and the anal sphincter, followed by microbial cultivation on the appropriate growth medium [4]. This method is not flawless due to its time-consuming nature, as the waiting time for the results is up to 7 days. The results themselves can also be ambiguous due to the often-difficult differentiation of GBS from other beta streptococci using phenotypic methods. It is a severe limitation especially in the case of advanced preterm labor, before the 36th week of pregnancy, i.e., prior to the recommended carrier screening test. Therefore, as an alternative for cultivation methods, in the latest CDC guidelines, mass spectrometry MALDI is also recommended for GBS detection. The main advantage of this method is a reduction in the waiting time for results as well as the possibility to distinguish *Streptococcus agalactiae* from other streptococci: *S. halichoeri* or *S. pseudoporcinus*, whose pathogenic role in newborn infection is not known [4]. When a positive result from a pregnant woman is obtained and GBS is detected in the studied specimen, introducing intrapartum antibiotic prophylaxis (IAP) is recommended. This procedure significantly reduced the morbidity in the case of early onset disease, however, IAP has not reduced the incidence of late onset disease, which may appear between the 7th and 90th day of life [7]. Additionally, the side effect of the comprehensive use of antibiotics is that is leads to the expansion of the adverse phenomenon of bacterial resistance to antibiotic therapy [4]. Moreover, this solution led to an increase in the rate of infections caused by Gram-negative bacteria [8]. As an answer to the limitations mentioned above and the unfavorable phenomena, scientists conduct extensive research to find alternative methods for GBS carriage detection in pregnant women. Novel diagnostic techniques should, among others, provide rapid and unambiguous results. An immunodiagnostic assay (i.e., ELISA) based on highly immunogenic and specific bacterial proteins (antigens) for detection of anti-GBS antibodies is being considered as a good candidate for innovative GBS carriage diagnostics. In our paper, we aimed to review some immunogenic proteins representative of *Streptococcus agalactiae*, which demonstrate features qualifying them as potential biomarkers in an innovative immunodiagnostic assay for the detection of GBS carriage and/or infection in pregnant women. It is worth underlining that such an assay could be useful also in the diagnostic of GBS infection in adults, in which this bacterium constitutes a growing clinical problem, taking the form of urinary tracts infection (UTI), sepsis, septic arthritis, meningitis and it is also isolated from diabetic foot/ulcer [9].

## 2. Main Text

The first immunological research on *Streptococcus agalactiae* was carried out by Professor Rebecca Lancefield who, in the 1930s, classified hemolytic streptococci into sera groups, according to the differences in the polysaccharide structure present in the bacterial cell wall. Thus, *Streptococcus agalactiae* was qualified into Group B, and it is described as GBS (Group B Streptococcus) [10]. Next, within group B, strains, according to the differences in the capsular polysaccharide (CPS) structure, were divided into three serotypes: I, II and III [10,11]. Currently, ten GBS serotypes: Ia, Ib, II-IX are distinguished, and their distribution is related to, among others, infection type, latitude, and age [2]. Since the 1970s, the number of publications describing specific GBS immunogenic molecules, with particular emphasis on immunogenic proteins successively grows from one year to another (source: https://pubmed.ncbi.nlm.nih.gov/ after entering the phrase: “immunogenic *Streptococcus agalactiae* proteins”, accessed on: 16 July 2021). Below we reviewed selected immunogenic *Streptococcus agalactiae* proteins, which can be considered as detective antigens in immunodiagnostic assay.

### 2.1. Alpha-like Protein 

**Figure 1:**
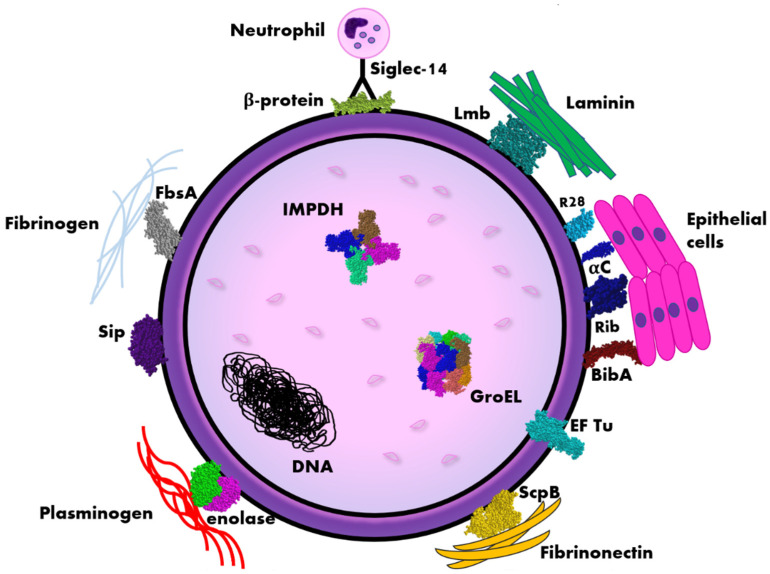
Scheme of the distribution of immunogenic proteins within the *Streptococcus agalactiae* (GBS) cell. Legend: αC, Rib, R28 (Alp3)—surface proteins belonging to Alp-like family, BibA—Group B streptococcus immunogenic bacterial adhesin, EF Tu—elongation factor thermo unstable, FsbA—fibrinogen-binding protein, IMPDH—inosine 5′-monophosphate dehydrogenase, Lmb—laminin binding protein, ScpB—streptococcal peptidase C5a, Siglec-14—sialic acid-binding immunoglobulin-like lectin, Sip—surface immunogenic protein. The diagram is not true to scale, the individual elements have a schematic dimension.

The best-known group of GBS proteins is the Alpha-like protein family (Alp), which include the following members: αC, Alp 1 (epsilon), Alp 2, Alp 3, Alp 4, and Rib. Alp proteins are conservative, chimeric and form mosaic structures on the GBS surface [12]. These surface-anchored proteins play an important role in *Streptococcus agalactiae* virulence, by supporting bacterial cell adherence to infected cells of the host (Figure 1). For example, αC protein mediates GBS invasion of cervical epithelial cells by interaction with glycosaminoglycan [13].

Alp proteins consist of a major signal peptide domain, a N-terminus region, comprising 170–180 amino acids, repeat area with numerous tandem repeats (8–10) of approximately 80 amino acids each, and a C-terminus region built from 40–50 amino acids. Their molecular masses may vary among particular GBS strains, for example α mass can range from 65 kDa to 165 kDa, and it can be explained by the differences in the number of repeats [14,15]. Amino acid sequences consisting of Alp family members are very likely, which may induce cross-reactivity among the individual proteins in the family.

Alp proteins are also described as immunogenic proteins [12]. In the context of immunogenicity, the best-known proteins are αC and Rib proteins. The immunogenicity of αC was described as the first immunogenic GBS protein. It was described for the first time in the 1980s on mouse model, when it was shown that purified αC protein isolated from *S. agalactiae* cell induced immunogenic response and protected mice against infection caused by these bacteria [16]. Afterward, this observation was confirmed in other studies [17,18,19,20]. This protein, together with β protein, form the so-called C antigen, which was detected in serum against the whole bacterial cell [21,22]. It participates in GBS pathogenesis, what was examined in the mouse model. It was demonstrated that the deletion of *bca* gene, which encodes αC protein, led to a reduction in bacterial virulence [19].

Another well-described immunogenic Alp-like protein is Rib, which demonstrates likeliness to αC, which was demonstrated by the analysis of the N-end amino acid sequence. Surprisingly, no cross-reactivity between these proteins was noticed, even though the nucleotide sequences are identical [23,24]. The Rib protein has been studied as a component of anti-GBS vaccine as the first Group B Streptococcus protein. The results received for a prototype recombinant Alpha-like protein subunit vaccine (GBS-NN), combined with α are promising. In a randomized placebo-controlled double-blind Phase 1 trial in healthy adult women, the safety and immunogenicity of GBS-NN vaccine was proved [25]. This also indicates potential usability of these proteins as biomarkers in immunodiagnostic detection of GBS carriage and infections caused by this pathogen.

The Alp3 protein, which can be also found under the name R28 in the literature, was first described for *Streptococcus pyogenes* (Group A Streptococcus) and its molecular likeliness to other Alp proteins was showed [26]. Analysis of the amino acid sequences of the R28 protein showed an identity of 98% between *S. pyogenes* and *S. agalactiae* species [27]. The Alp3 protein is considered to be a chimera of three *S. agalactiae* proteins: α, β and Rib [28].

As it was described above, immunoreactivity of the Alp-like proteins had been proved. Additionally, their conservation allows to consider them as good candidates for markers in GBS immunodetection. Nevertheless, the distribution of individual Alp proteins within the species could be a limiting factor as long as only one Alp protein is present in a single GBS strain [29]. Therefore, constructing an immunoassay based on a single Alp protein can be insufficient and use of all Alp proteins should be considered.

### 2.2. β Protein

β protein (approx. 130 kDa), also called Bac, had been previously described as a component, together with αC, of antigen C, belonging to the Alp-like family. Although both proteins are encoded by genes located nearby on the chromosomes, they do not demonstrate a close relationship [15,17,30]. However, β indicates homology to the Alp3 protein, and it consists of 1159 residues [28,31]. The distinguishing feature of this protein, compared to most of the surface proteins representative of Gram-positive cocci, is lack of long repetitive tandem sequences [32]. Although the virulent nature of this protein has not been fully described yet, it is hypothesized that, due to its ability to bind to elements of the human immune system (Figure 1, Table 1), it may also be associated with virulence [33]. In addition, the β protein has an affinity for two components of the human immune system: the Fc fragment of IgA antibodies and the H factor, which regulate the alternative pathway of complement activation, so that its action is directed against the infecting pathogen, not human cells or tissues. This fact may suggest an important role of this protein in induction of immunity [15]. It was also showed that β protein binds to sialic acid-binding immunoglobulin-like lectin 5 (Siglec-5), which is an inhibitory receptor for phagocytosis, and therefore attenuates innate immune responses in the infected organism, and promotes bacterial survival [34]. Moreover, GBS β protein binds to Siglec-14 on neutrophils, and this engagement counteracts the host immune suppression induced by pathogen by activation of p38 mitogen-activated protein kinase (MAPK) and AKT signaling pathways. It is also worth to underline that Siglec-5 and Siglec-14 expression has been present in amniotic epithelium, which is the place of the initial contact of *S. agalactiae* with the fetus [35].

In the experiment based on active immunization with β protein of pregnant mice, it was shown that offspring did not develop infection after contact with GBS strains containing the β protein [36]. In addition, it has been shown that IgG anti-β antibodies can cross the placenta, which may indicate the possibility of mother-to-child transmission of immunity, and thus offer protection against GBS infection [37]. Immunogenicity in the presence of IgM and IgG class antibodies was studied by natural exposure of pregnant women to β protein. Geometric mean concentration of anti-β C protein IgM and IgG antibodies was measured in an enzyme-linked immunosorbent assay (ELISA). The research was carried out on 16 pregnant women colonized with GBS, and 48 uncolonized match-age pregnant women, who constituted the control group. In the study group, 3 out of 16 women demonstrated a significant growth in IgM and IgG antibodies; therefore, it had been concluded that GBS invasion, but not colonization, induces an increase in antibody titer [38]. In summary, with no doubt, immunogenicity of β protein had been shown, however, due to insignificant growth of the antibody concentration in carriers, usability of this protein as a marker in immunoassay could be limited to detection of GBS infection but not carriage.

### 2.3. Lmb Protein

Laminin binding protein (Lmb, LmbP) belongs to lipoproteins by its molecular weight of 43 kDa and it is exposed on the cell surface of most *Streptococcus agalactiae* strains (Figure 1, Table 1). The Lmb protein, consisting of 306 amino acids, shows homology to the members of the Lra1 protein family, which are known for their role in adhesion and metal transportation in Gram-positive bacteria. Lmb is involved in colonization of the host and invasion through damaged epithelial cells [39,40]. Even though the Lmb name was limited to *Streptococcus agalactiae* species, an almost identical protein of *Streptococcus pyogenes* has been referred to as Lsp or Lbp [41,42]. For both species, the gene encoding this protein is located above the C5a-peptidase encoding gene and nucleotide sequence identity between these two species is >98% in this region. Nevertheless, contiguous sequences in the two genomes show no homology, which may indicate that the region was horizontally transferred [41,43]. The immunogenic character of Lmb had been studied for *Streptococcus pyogenes* (GAS), which causes various diseases ranging from pharyngitis to severe infections such as a toxic shock-like syndrome and necrotizing fasciitis, and at some points, is phylogenetically similar to *Streptococcus agalactiae* [44,45]. A recombinant Lmb GAS protein (rGAS-Lmb) had been studied in the presence of serum from patients with rheumatic fever and individuals with uncomplicated streptococcal infections. Antibody response for the study and control groups was examined by ELISA assay, and the differences observed in reactivity were significant, whereas no difference between infection types was noticed [46]. No data for Lmb immunoreactivity for the studied group of pregnant women colonized by *Streptococcus agalactiae* is available, however it can be assumed that, according to its confirmed immunoreactivity for *Streptococcus pyogenes*, it may also be considered as a potential candidate for GBS detection.

### 2.4. Sip Protein

Sip protein (surface immunogenic protein) with weight of 53 kDa was, in opposition to other previously described surface proteins, identified following immunological screening of a genomic library. Sip is present in all GBS strains, regardless of the serotype represented (Figure 1, Table 1). Analysis of the nucleotide sequences for the studied strains confirmed their 98% identity of the *sip* gene, which encodes the Sip protein. It indicates the conservation of this 434 amino acid protein. Moreover, Sip is also described as an immunogenic protein. Immunization in mice with the recombinant Sip protein demonstrated efficient protection against severe consequences of GBS infection caused by strains representing six serotypes (Ia, Ib, II, III, V, and VI) [47]. Immunogenicity of the Sip protein was also studied in the context of the oral vaccine administrated to tilapia, a fish species in which infection caused by *Streptococcus agalactiae* is common and leads to huge financial losses in fishery. The immunizing protein against *Streptococcus agalactiae* was expressed in *Bacillus subtilis* spores. It was shown that immunization indicated an effective immune response and provided protection against GBS infection [48,49]. Another promising result was obtained for the investigation of a decrease in the *S. agalactiae* colonization in a mouse model following oral administration of the vaccine based on Sip protein [50]. This may suggest that Sip protein induces cross-protective immunity against GBS infections, therefore, it can be considered as a potential vaccine candidate; on the other hand, its conservation qualifies the Sip protein as a potential candidate for immunodiagnostic assay. Research on an immunodiagnostic assay based on Sip protein as a detection antigen has already been carried out. Nevertheless, selectivity examined in an indirect ELISA assay for bovine mastitis detection reached 75.6% (for 45 studied serum antibodies isolated from cows, 35 were positive, whereas control examination performed by PCR gave 100% positive results) [51]. Immunoreactivity of the Sip protein was also studied as an element of fusion protein combined with two other membrane surface-associated GBS proteins, which were fibronectin (FbsA) and phosphoglycerate kinase (Pgk) in the indirect ELISA assay for detection of bovine mastitis. The obtained results indicated relatively higher sensitivity in comparison with mono-antigen fusion protein Sip [52]. Immunogenicity of the Sip protein, according to its conservation, was also investigated in the context of usage for monoclonal antibody generation to develop immunochromatographic test kit for GBS detection in pregnant women [53]. Another study was focused on examination of Sip protein as a biomarker in a rapid immunochromatographic test for detection of Group B streptococcus colonization in vaginal and/or rectal tracts in pregnant women during the 35th–37th weeks of pregnancy. The obtained results were very promising, and the developed test was characterized by high specificity, with selectivity reaching respectively 93.1% and 100% [54]. Therefore, we conclude that the Sip protein can be doubtlessly considered as a GBS detection antigen.

### 2.5. BibA Protein

BibA protein (Group B Streptococcus immunogenic bacterial adhesin) is an immunogenic bacterial adhesin, exhibiting molecular weight of approx. 80 kDa, which demonstrates antiphagocytic properties. BibA mediates GBS adherence to both human cervical and lung epithelial cells (Figure 1, Table 1). The protein consists of the N-end α-helix rich domain, proline-rich region, LPXTG cell-wall anchoring motif, and is composed of 594 amino acids. Due to its N-terminal helical domain, which consists of three antiparallel α-helical-bundle motifs, it is considered as unique, and thus is qualified to a new class of Gram-positive surface adhesins [55]. This protein is identified on the surface of *S. agalactiae* strains, but interestingly, it is also present in GBS culture supernatants [56,57]. Four allelic variants (I, II, III, IV) of this protein correlated with serotypes had been described, and what is worth underlining, variant IV, which demonstrated high likeliness to the bovine counterparts, was exclusively associated with the highly virulent ST-17 GBS strain. Therefore, BibA is considered as a multifactorial GBS virulence factor [7,58,59]. BibA expression is modulated by the CovS/CovR 2-component regulatory system, and it specifically binds to C-4 binding protein in humans, which is a regulator of the classical complement pathway. It has been demonstrated that deletion of *bib*A gene resulted in reduced capacity of GBS to survive in human blood as well as decreased ability to resist opsonophagocytic killing by human neutrophils. Additionally, BibA expression led to an increase in the GBS virulence in a mouse model [56,57]. While mice immunization with a recombinant BibA protein (GBS-V BibA) conferred immunity and protected them from vaginal colonization by *S. agalactiae*, and eventually led to decrease in mortality. Antibody response after immunization was examined in ELISA assay in which plates were coated with BibA protein. This indirect use indicates the usability of BibA protein as detection antigen in ELISA for GBS carriage and/or infection diagnosis [60]. A similar conclusion can be drawn from the research whose aim was to examine the association between antibodies against *Streptococcus agalactiae* surface proteins and recto-vaginal colonization during pregnancy, in which titers of IgG antibodies were measured in Luminex multiplex immunoassay [61].

### 2.6. FsbA Protein

Fibrinogen-binding protein (FsbA), approx. 26 kDa, is one of the virulence factors of *Streptococcus agalactiae*, and its role is the attachment to fibrinogen, which leads to fibrinogen-dependent aggregation of platelets (Figure 1, Table 1). It was demonstrated that GBS mutants lacking *fsb*A gene lost the aggregation ability. Furthermore, application of monoclonal anti-FsbA antibodies impeded bacterial binding to fibrinogen as well as platelet aggregation caused by *Streptococcus agalactiae* [62,63,64]. FsbA is composed of 16 amino acid repetitive units. It has been demonstrated that human fibrinogen was bound by the repetitive protein region, and even a single repeat had the ability attach to fibrinogen [63]. FsbA protein consists of C-terminus cell wall anchoring motif (LPKTG), which indicates that this protein is covalently attached to the cell wall. The second GBS fibrinogen binding protein is FsbA’s analogue—FsbB, even though these proteins do not reveal significant likeliness to each other. The feature distinguishing FsbA from other fibrinogen binding proteins, representative to other bacterial species, is the LPKTG motif [65]. As long as FsbA protein structure is well described, its function is barely known, except its immunogenic role. It was shown that maternal immunization of mice with 6pGST, a protein fragment which consists of five repeats, significantly protected the offspring against lethal infection caused by *Streptococcus agalactiae*. It was demonstrated that the protective role of the antibodies can be obtained by administration of anti-6pGST serum from adult animals. The introduction of serum with antibodies led to protection by bacterial opsonophagocytosis or resulted in neutralization of FbsA-mediated Fng binding. Two-track action had also been noticed [66]. Other studies showed RPS (relative percentage survival) value after tilapia vaccination consisting of GBS FsbA protein reached 40.63% [67]. It allows to define FsbA as a multifunctional immunogenic protein, including immunoprotection as well as activation of the innate immune responses in the host and relevant antibody responses [68].

### 2.7. ScpB Protein

Streptococcal peptidase C5a (ScpB) is an enzymatic surface protein, which belongs to serine protease and is related to the subtilisin family of enzymes (Figure 1, Table 1). It was demonstrated that substitution of the following amino acids: Ser512, His193, or Asp130 with alanine confirmed their proteolytic role, which has an impact on the whole protein [69]. This protein is a large molecule, which consists of over 1100 residues, excluding the signal sequence and it weighs over 126 kDa [70,71]. A comparison of the amino acid sequence between Scp N-terminal catalytic triad and subtilisin revealed homology [69]. C-terminus of Scp includes the peptidoglycan anchor sequence, which is common to other Gram-positive bacterial surface proteins [72]. C5a peptidase is responsible for inactivation of one of the components of the human complement, i.e., decay product of C5, C5a converting enzyme [73]. C5a peptidase was first described for *Streptococcus pyogenes*, however, further studies have shown the presence of this enzyme also in *Streptococcus agalactiae* strains. Sequence analysis of both proteins revealed their 95% similarity, which is a consequence of horizontal gene transfer between both species. Therefore, in order to distinguish between them, the following nomenclature is used: C5a peptidase for the species *Streptococcus pyogenes*, belonging to the serological Group A, is called ScpA, while analogously for *Streptococcus agalactiae*, representative for Group B, this protein is described as ScpB [70,72,73,74,75,76]. Interestingly, *scp*B gene is common for GBS strains isolated from humans, whereas, in bovine, it is barely present [41,77]. Additionally, ScpB-related cell envelope proteases, which have a multi-domain structure, are also common to lactic acid bacteria [70,77].

C5a peptidase is described for its virulence function. It is responsible for disrupting neutrophil recruitment, resulting in a reduction in the inflammatory response elicited in the infected tissue. Interestingly, inhibition of neutrophil recruitment had not been observed in the mouse model [78,79]. This non-obvious observation should be considered during the selection of an appropriate model for research on the GBS vaccine, the component of which will be C5a peptidase, as long as Scp reveals its immunogenic character [15,80,81]. Another Scp feature, on the basis of which it is qualified as a virulence factor is the ability to bind fibrinogen, which may promote bacterial adhesion to epithelial and endothelial cells [15,82]. However, studies with mutant bacterial cells, lacking ScpB, revealed only a partial reduction in the fibrinogen binding capacity compared to the unmutated strains. It can be explained by the fact that Group B streptococci possess other proteins that are able to bind fibrinogen [83].

As it was mentioned above, both ScpA and ScpB also show strong immunogenic properties, and due to their conservation, they are considered as good vaccine candidates [84,85]. Research on mice immunized with the purified ScpB protein, which were next infected with *Streptococcus agalactiae* strains via the nasal route, showed a higher clearance of bacteria from the lungs [86]. Moreover, experiments on hyperimmune rabbit serum showed opsonizing activity with mouse macrophages and human whole blood. It has also been suggested that antibodies directed against ScpB antigen may protect from infection by disrupting fibrinogen binding [85]. Due to the features described above, ScpB can be considered as a biomarker in an immunodiagnostic assay, and its usability had previously been demonstrated in the enzyme-linked immunosorbent assay [87,88].

### 2.8. Enolase

Enolase is a dimeric protein, weighing approximately 47 kDa (approx. 430 aa), which is a glycolytic enzyme and catalyzes the penultimate stage of glycolysis, which is the dehydration reaction of 2-phosphoglycerate to phosphoenolpyruvate [89,90]. Enolase is also very conservative among the Streptococcus species. It had been demonstrated that identity of amino acid sequences exceeded 90% among the following species: *Streptococcus pneumoniae*, *Streptococcus agalactiae*, *Streptococcus sobrinus*, and *Streptococcus mutans* [91,92]. Furthermore, likeliness between human and streptococcal enolase reaches 49% [93]. There are three distinct isoforms of enolase: α, β, and γ, with the same molecular mass each. Nevertheless, the isoform typically found in bacteria is α-enolase. Enolase is a common protein for numerous bacterial species, and it plays an important role in the pathogenesis by binding plasminogen on the surface of the host cell, which mediates fibrinolysis, homeostasis, and the degradation of the extracellular matrix (Figure 1, Table 1) [94,95]. Interestingly, the investigation of amino acid sequences of the α-enolase in *Streptococcus mutans*, which is responsible for inducing dental caries, revealed a lack of the hexameric cell wall anchoring motif (LPXTGX) [96]. Thus, the way of transportation and cell wall attachment is not well understood yet [91]. This protein has also been described in the context of its immunogenicity for *Streptococcus agalactiae* [84,97,98,99,100,101]. It has been shown that streptococcal anti-enolase antibodies may react with human enolase produced after *S. pyogenes* infection [93]. Additionally, antibodies directed against enolase had been detected in some autoimmune diseases, such as systemic lupus erythematosus, mixed cryoglobulinemia, systemic sclerosis, and rheumatoid arthritis [102]. In our previous paper, we decided to take the next step and detected epitopes representative of enolase, which specifically recognized anti-GBS IgG antibodies, and which were studied in ELISA with the presence of umbilical cord blood serum from pregnant women qualified as GBS carriers. Presently, these epitopes are being investigated as potential components in an immunodiagnostic assay for detection of GBS carriage and/or infections in pregnant women [103]. Nevertheless, consideration of enolase as a detection antigen requires taking into account the fact of relatively close sequence similarity among streptococci as well as a possibility of cross-reactivity with human enolase.

### 2.9. Elongation Factor Tu

Elongation factor thermo unstable (EF Tu) is a moonlight protein, weighing approx. 44 kDa and consisting of approx. 400 residues, which is involved in several pathogenic functions, such as: adhesion, invasion, and modulation of the host immune system through stimulation of humoral immune response [104,105,106,107,108,109]. This conservative protein is the most abundant protein in bacteria, it constitutes up to 5% of the total cell content, and it is common for both prokaryotic and eukaryotic organisms [108,110]. In the bacterial cell, EF Tu constitutes part of the membrane cytoskeleton, and it is involved in the elongation phase of protein synthesis as well as in the translation process in prokaryotic cells (Figure 1, Table 1). As a GTPase, it ensures a catalysis reaction, whose aim is the correct addition of the consecutive amino acid to a growing nascent polypeptide chain [111]. Even though EF Tu is a component of the membrane cytoskeleton, it does not possess a signal secretion motif, due to its moonlight roles, it requires to localize on the cell surface [108,110,112,113]. Probably the protein is exported by several paths, such as cell lysis, extracellular vesicle secretion or via association with proteins that are secreted by the Sec machinery 54 [114,115,116]. It plays an important role in shuttling aminoacylated tRNAs to the ribosome during protein translation [104,117,118,119,120]. In *Escherichia coli*, EF Tu consists of three domains, whereas the first domain forms a helix structure with the Rossmann fold topology, which is characterized by tertiary protein fold composed of both α-helical and β-strands that bind nucleotides [121], the second and the third domains mostly consist of β-sheets [111,122]. Amino acid sequences of the EF Tu protein are characteristic of various bacterial species representing similarity in sequence identity; therefore, they have been used to generate a phylogenetic tree [123]. This moonlight protein interacts with several molecules, such as CD21, factor H, fibrinogen, fibronectin, laminin, nucleolin, tachykinin, plasminogen and several complement factors [107,124,125,126,127]. EF Tu is also known for its immunogenic role. Antibodies against EF Tu are detected after infections caused by *Burkholderia pseudomallei*, *Chlamydia trachomatis*, *Mycoplasma capricolum*, *Mycoplasma hyopneumoniae*, *Mycoplasma ovipneumoniae*, and *Staphylococcus aureus* [128,129,130,131,132,133]. On the other hand, EF Tu for *Haemophilus influenzae* is recognized by antibodies, which mediate innate immunity of the host against NTHi [134]. While, when GBS it found, the protein is common for all *Streptococcus agalactiae* strains, regardless of molecular differences, such as the serotypes represented, genes encoding Alp proteins or the origin [101,135,136]. Research on a subunit anti-GBS vaccine based on EF Tu revealed the capability of the indication of the immunity and protection of tilapia against infection caused by *Streptococcus agalactiae*, however vaccination with the subunit EF Tu vaccine generated moderate immune protection with RPS equal 70% [136]. Mouse vaccination against EF Tu of *Streptococcus pneumoniae* resulted in the animals’ protection against lethal challenges, and on the molecular level, increased cytokine, IgG1 and IgG2a, and CD4+ T-cell production was observed [137]. Promising results of rEF-Tu vaccination was obtained for fish as well [136]. In our previous paper, similarly to enolase described above, we aimed to detect the highly specific epitopes representative of GBS EF Tu as potential markers in an immunodiagnostic assay for detecting GBS carriage in pregnant women, and the obtained results were very promising [135]. However, it is necessary to consider some limitations, such as a tendency for cross-reactivity between different bacterial species with special emphasis on unencapsulated species, among others, various Gram-positive streptococci of the oral microbiome. Pneumococci are not detectable, what is related to the capsules of pneumococci and meningococci, which shield surface-associated EF Tu from antibody detection [134,138].

### 2.10. IMPDH

Inosine 5′-monophosphate dehydrogenase (IMPDH, dehydrogenase IMP), with a mass of 53 kDa, is a stable purine, which participates in catalysis of the key stage of de novo synthesis of the guanine and adenine nucleotides in all organisms, in other words, it is a crucial precursor for DNA and RNA synthesis [103,139,140]. IMPDH monomers contain 400–500 amino acids, and these differences depend on the presence of a subdomain that is not required for enzymatic activity. Each monomer is built of two domains: the α and β (α/β)8 barrel catalytic domain and the subdomain containing two CBS domains (cystathionine beta synthase, Bateman domain) [140]. The protein most often takes the form of tetramers, and it is the most stable configuration (Figure 1, Table 1) [141,142,143]. The IMPDH tetramers have square planar geometry, with the sides of the barrels at the subunit interfacing and CBS domains sticking out from the corners of the tetramer. The junction between domains is flexible, and the relative orientation can vary [144]. The IMPDH also catalyzes conversion of IMP to XMP as the first committed and rate-limiting step in guanine nucleotide biosynthesis. In turn, XMP is subsequently converted to GMP by the action of GMP synthetase (GMPS). This IMPDH/GMPS pathway is common for almost all organisms. IMPDH is also involved in glycoprotein synthesis, energy transfer, signal transduction in cells, and NAD-dependent catalysis. It is worth to mention that there are many genes encoding IMPDH [140]. In the literature, due to its crucial role in cell replication, IMPDH is frequently described as a potential target in antiviral, antibacterial and anticancer therapies. It is also considered as a part of autoimmunological disease treatment [140,145,146,147]. Unfortunately, it has been reported that some pathogens developed resistance to IMPDH inhibitors by amplifying the *IMPDH* gene, which significantly limits its usability as an antibiotic [140]. This protein also demonstrates immunogenic features in some bacterial species [148,149,150,151]. Immunoreactivity to *Streptococcus agalactiae* was also showed. Moreover, highly specific IMPDH GBS epitopes recognized by umbilical cord blood isolated from GBS-positive women were identified, and what is worth to underline, it was performed for the first time [101,103]. The obtained results allowed to consider them as potential antigens in an immunodiagnostic assay for GBS carriage detection.

### 2.11. GroEL

GroEL is a 57 kDa protein, which belongs to the chaperonin family. Structurally GroEL is a double ring tetradecamer, composed of seven identical 10 kDa subunits in cis and trans positions, which demonstrates ability to form barrel-like structures with hydrophilic cavities, which are isolated from each other by the equatorial domains and the C-terminal tails of each subunit (Figure 1, Table 1). Each oligomeric complex consists of fourteen identical 57kDa subunits, and each subunit can be divided into three domains: apical, intermediate, and equatorial. Apical domains are located at the outer edge of the rings and contain binding sites for GroES, with which they frequently form complex and non-native proteins. While equatorial domains adjoin each other within the individual ring, and they consist of a domain with the ATP binding site. Intermediate domain is connected with apical domain through the slender intermediate domain [152,153,154]. This protein is common for numerous bacteria, and, moreover, it is structurally and functionally closely related to the human heat shock protein (Hsp60) [155]. The key role of GroEL is folding de novo emerging proteins. Its functions, as a chaperonin protein, are recognizing, binding, and releasing other proteins. These actions are performed due to the characteristic structure of the apical domain, which consists of non-polar amino acids on the inner surface, and hydrophobic external surface, which demonstrates the ability to capture and tightly bind protein folding intermediates [156,157]. GroEL participates in folding of a wide range of proteins, with special emphasis on these, which are typically large (>20 kDa), slow folding, and prone to aggregation [158,159].

GroEL is also described as a virulence factor and participates in pathogenesis, even though the switching mechanism from folding supporting protein to virulence factor is not yet understood [160,161]. Virulence of GroEL can be carried out by promoting infection by replication and persistence followed by adhesion, invasion, evasion of host immune responses, and modification of host cell responses [162]. GroEL is described as a moonlight protein; nevertheless, its ability to change roles has not been determined yet [163]. As an example of moonlighting, GroEL from some species, such as: *Lactobacillus johnsonii*, *Mycoplasma pneumoniae* or *Salmonella enterica*, displays an ability to support adhesion to mucin [164]. Mucin belongs to glycoproteins, which create a gel-like layer on the mucosal surface, and thus protect the host from pathogen colonization and invasion [165]. Therefore, binding bacterial cells throughout GroEL protein to mucin promotes initiation of the colonization and invasion [163]. In turn, GroEL from *Helicobacter pylori* promotes binding iron, which is essential for growth of this species, and this ability could allow it to compete with other species for a colonization niche [166,167]. Another interesting and surprising function of GroEL is its toxic effect on some species of insects. For example, GroEL produced and excreted by *Enterobacter aerogenes* paralyzed a cockroach but not mice [168]. Some research suggest that GroEL can also be considered as a potential plant protection product, because it had been showed that GroEL from *Xenorhabdus nematophila* expressed in tobacco induced resistance to invading insects [169]. Even though GroEL plays its role inside a cell, it has an ability to relocate on the cell surface. As a stress response protein, it demonstrates an ability to introduce changes in the level of expression as well as in the cellular localization from the cytosol to the cell surface or the secretome. Therefore, apart from folding, GroEL is also an immunogenic protein, because it can be presented to the antibodies [163]. Mouse vaccination with rGroEL from *H. pylori* induced immune protection for both mother and infants [170]. Successful immunization with rGroEL had also been demonstrated for other species such as: *Mycobacterium bovis* [171] and *Lawsonia intracellularis* [172].

This molecular chaperon, as well as its epitopes, have an ability to specifically bind with anti-GBS antibodies present in both umbilical cord and vascular blood [103]. Immunogenic features of GBS GroEL were also investigated for tilapia. Immunization with recombinant GroEL induced to increase titers of anti-rGroEL antibodies and insure protection against *Streptococcus agalactiae* with RPS on an approximate level of 70%. While testing the titer of antibodies produced after rGroEL immunization, an ELISA in which detection antigen constituted rGroEL protein was performed [49]. It indirectly confirms the usability of this protein as a biomarker in an immunodiagnostic assay.

## 3. Conclusions

We conclude that the proteins described above, with special emphasis on Sip, BibA, Lmb, FsbA, ScpB IMPDH, and GroEL, due to their proven immunoreactivity and conservation, can be considered as good candidates in immunodiagnostic assays for detection of GBS carriage and infection particularly in pregnant women, but, with high probability, also in other patients in the so-called high-risk groups, such as elderly or immunosuppressed adults. Consideration of Alp proteins and the remaining three proteins, which are β-protein, enolase, and elongation factor Tu, as potential detection antigens should take into account their limitations, such as lack of universality in the case of Alp proteins, risk of cross-reactivity among other bacterial species as well as human counterparts for enolase, and EF Tu, or insignificant growth of the antibody concentration in carriers following immunization with β protein. We also believe that in the near future many more immunogenic proteins will be described and studied, and this knowledge will find its practical application, i.e., in the ELISA assay or immunochromatographic assay investigation.

## Figures and Tables

**Table 1:** Summary of chosen GBS immunoreactive proteins—potential biomarkers in immunodiagnostic assays for detection of GBS carriage/infection and components of a vaccine against *Streptococcus agalactiae* infections.

Proteins’ Name	Molecular Mass	Function/Characteristic	Immunoreactivity	Localization in Cell
Alpha-like proteins: -αC, -Alp 1 (epsilon) -Alp 2 -Alp 3 (R28), -Alp 4, -Rib	65 kDa–165 kDa	Facilitation of GBS adherence to epithelial cell by interaction with glycosaminoglycan Participation in pathogenesis	First described immunogenic GBS protein—in mouse model (αC) in 1980 Component of the so-called C antigen (αC) Component of prototype of anti-GBS vaccine (GBS-NN) – 1 phase of clinical trials (αC, Rib)	Surface anchored
β-protein (Bac)	130 kDa	Ability to bind to elements of the human immune system, may indicate its virulence Attenuates innate immune responses, and thus promotes bacterial survival by binding to sialic acid-binding immuno-globulin-like lectin 5 (Siglec-5) Counteracts the host immune suppression by binding to Siglec-14 on neutrophils	Component of the so-called C antigen Immunization of pregnant mouse with β-protein protected infants from GBS infection Proven ability of IgG anti-β to pass through the placenta Proven growth of IgG and IgM in pregnant women after exposure to GBS β protein	Cell surface
Laminin binding protein (Lmb, LmbP)	43 kDa	Involved in colonization of the host and invasion through damaged epithelial cells	Immunogenicity of Lmb was shown for GAS, thus its immunoreactivity for GBS is hypothesized	Cell surface
Surface immunogenic protein (Sip)	53 kDa	Unknown	Identified following immunological screening of a genomic library Proved mouse protection against GBS infection representing 6 serotypes Proved tilapia protection against GBS by oral vaccination Proved mouse protection against GBS by oral vaccination 75.6% sensitivity of indirect ELISA for bovine mastitis diagnostic Component of fusion protein in the indirect ELISA assay for detection of bovine mastitis Studied in the context of monoclonal antibody generation to develop immunochromatographic test kit for GBS detection in pregnant women Promising biomarker in a rapid immunochromatographic test for GBS detection in pregnant women	Cell surface
Group B Streptococcus immunogenic bacterial adhesin (BibA)	80 kDa	Demonstrates antiphagocytic properties Mediates GBS adherence to both human cervical and lung epithelial cell Multifactorial GBS virulence factor Binds to C-4 binding protein in humans	Mice immunization with a recombinant BibA protein (GBS-V BibA) protected from vaginal colonization with GBS Promising biomarker in indirect ELISA for GBS carriers/infection diagnosis Promising biomarker in Luminex multiplex immunoassay for GBS detection	Cell surface
Fibrinogen-binding protein (FsbA)	ap. 26 kDa	Promotes GBS attachment to fibrinogen Activates the innate immune responses in the host and relevant antibody responses	Proved protection of mice infants against GBS by pregnant mother immunization Proved protection by bacterial opsonophagocytosis or neutralization of FbsA-mediated Fng binding by administration of serum with anti-FsbA antibodies Proved tilapia protection after immunization	Cell surface
Streptococcal peptidase C5a (ScpB)	126 kDa	Responsible for inactivation of one of the components of the human complement Responsible for disrupting neutrophil recruitment Demonstrates the ability to bind fibrinogen, thus promotes bacterial adhesion to epithelial and endothelial cells	Nasal immunization of mice with purified ScpB protein demonstrated higher bacterial clearance from the lungs Proved opsonizing activity with mouse macrophages and human whole blood in experiments on hyperimmune rabbit serum It has been hypothesized that antibodies directed against ScpB antigen may protect from infection by disrupting fibrinogen binding Proved usability of ScpB in ELISA	Cell surface
Enolase	47 kDa	Catalyzes the penultimate stage of glycolysis, which is the de-hydration reaction of 2-phosphoglycerate to phosphoenolpyruvate Plays an important role in the pathogenesis by binding plasminogen on the surface of the host cell	Anti-enolase antibodies can be detected in certain autoimmune diseases Enolase epitopes considered as potential biomarkers in immunodiagnostic assay	Cell wall
Elongation factor thermo unstable (EF Tu)	44 kDa	Plays an important role in the pathogenesis by promoting adhesion, invasion, and modulation of the host immune system through stimulation of humoral immune response Involved in the elongation phase of protein synthesis as well as in the translation process in prokaryotic cells Ensures a catalysis reaction Plays an important role in shuttling aminoacylated tRNAs to the ribosome during protein translation Interacts with several molecules, such as CD21, factor H, fibrinogen, fibronectin, laminin, nucleolin, tachykinin, plasminogen and several complement factors	Anti-EF Tu antibodies are being detected after infection caused by several pathogen species Vaccine against GBS based on EF Tu is being studied for tilapia Mouse vaccination with rEF Tu of S. pneumoniae led to increased numbers of cytokine, IgG1 and IgG2a, and CD4+ T-cell Epitopes of GBS EF Tu are being investigated as potential biomarkers in ELISA assay for carrier/infection diagnosis	Part of membrane cytoskeleton, cell surface
Inosine 5′-monophosphate dehydrogenase (IMPDH)	53 kDa	By participation in catalysis of the key stage of de novo synthesis of the guanine and adenine nucleotide considered as crucial precursor for DNA and RNA synthesis Catalyzes conversion of IMP to XMP Involved in glycoprotein synthesis, energy transfer, signal transduction in cells, and NAD-dependent catalysis Potential target in antiviral, antibacterial and anticancer therapies Considered as a part of autoimmunological disease treatment	Proved immunoreactivity of IMPDH epitopes in the presence of human umbilical cord blood	Intracellular
GroEL	57 kDa	Structurally and functionally closely related to the human heat shock protein (Hsp60) Plays a key role in folding de novo emerging proteins (recognizing, binding, and releasing other proteins) Participates in folding of a wide range of proteins, with special emphasis on these, which are typically large (>20 kDa), slow folding, and prone to aggregation Known as a virulence factor and it participates in pathogenesis, however mechanism is unknown GroEL may promote infection by replication and persistence followed by adhesion, invasion, evasion of host immune responses, and modification of host cell responses GroEL can be considered as potential plant protection products	Promising results of vaccination of pregnant mice against several bacterial species Promising results of anti-GBS vaccines administrated to tilapia Proved immunoreactivity of GroEL epitopes in the presence of human umbilical cord blood and vascular blood	Cytosol, cell surface, secretome
